# Chitosan–Gelatin Scaffolds Loaded with Different Antibiotic Formulations for Regenerative Endodontic Procedures Promote Biocompatibility and Antibacterial Activity

**DOI:** 10.3390/jfb15070186

**Published:** 2024-07-04

**Authors:** Maha Alghofaily, Aljowhara Almana, Jenan Alrayes, Rhodanne Lambarte, Michael D. Weir, Fahd Alsalleeh

**Affiliations:** 1 Restorative Dental Sciences, College of Dentistry, King Saud University, P.O. Box 60169, Riyadh 11545, Saudi Arabia; 2 College of Dentistry, King Saud University, P.O. Box 60169, Riyadh 11545, Saudi Arabia; aljowharaalm@gmail.com (A.A.); jenan_r@hotmail.com (J.A.); 3 Molecular and Cell Biology Laboratory, King Saud University Medical City, P.O. Box 60169, Riyadh 11545, Saudi Arabia; rlambarte@ksu.edu.sa; 4 Department of Biomaterials and Regenerative Dental Medicine, University of Maryland School of Dentistry, Baltimore, MD 21201, USA; michael.weir@umaryland.edu

**Keywords:** regenerative endodontics, intracanal medicaments, modified triple antibiotic paste, root canal disinfection, chitosan scaffold

## Abstract

Background: This study investigated the biocompatibility and antibacterial efficacy of chitosan–gelatin (CH-G) scaffolds loaded with slow-releasing antibiotic formulations used in regeneration endodontic procedures (REPs). Methods: Scaffolds were fabricated using freeze drying and loaded with varying concentrations of augmentin or modified triple antibiotic paste (mTAP). High-resolution scanning electron microscopy (SEM) was used to characterize the scaffold, while drug release was monitored via UV-Vis spectrophotometry. Immortalized human mesenchymal stem cells (hMSCs) were cultured on CH-G scaffolds alone (control), either 0.1 mg/mL or 1 mg/mL of augmentin or mTAP, and 10 mg/mL calcium hydroxide (Ca(OH)_2_). Cell viability and proliferation were assessed using the Alamar Blue assay and SEM, respectively, and live/dead staining further corroborated cell viability. Antibacterial activity against Enterococcus faecalis was evaluated using the MTT assay and confocal laser scanning microscopy (CLSM). Results: Augmentin at 0.1 mg/mL appeared to promote better cell growth and attachment within the scaffolds than all other formulations, exhibiting acceptable viability. SEM revealed improved cell attachment in augmentin and mTAP groups compared to the Ca(OH)_2_ group. Augmentin at 1 mg/mL and mTAP groups significantly reduced viable bacteria compared to controls. Augmentin groups and mTAP at 1 mg/mL were highly effective in eliminating *E. faecalis* biofilms, with mTAP potentially causing more cell death within the remaining biofilm structures. Conclusions: This study suggests that CH-G scaffolds loaded with augmentin and mTAP, particularly at a concentration of 1 mg/mL, offer promising advantages for REPs due to their biocompatibility, antibacterial efficacy, and ability to promote cell attachment. Further research may explore the long-term effects in clinical settings.

## 1. Introduction

Pulpal necrosis and subsequent endodontic infection in immature teeth present a significant clinical challenge. These conditions can halt root development, compromising the long-term strength and prognosis of the affected tooth [1]. Regenerative endodontic procedures (REPs) offer a promising approach to treating necrotic pulp in immature permanent teeth. REPs aim to stimulate a host response against bacteria while promoting root development and increased dentin formation [2,3,4]. This approach aligns with the principles of tissue engineering, potentially by introducing stem cells, growth factors, and scaffolds into the devitalized root canal system.

Persistent infection remains a primary hurdle to successful REP outcomes [5]. Therefore, effective root canal disinfection using intracanal irrigants and medicaments is critical. These methods reduce microbial biofilms within the root canal system and optimize treatment success [2,6].

The American Association of Endodontists (AAE) recognizes various intracanal medicaments for REP treatment, including triple antibiotic paste (TAP), double antibiotic paste (DAP), modified triple antibiotic paste (mTAP) incorporating clindamycin, amoxicillin, or cefaclor instead of minocycline, and calcium hydroxide (Ca(OH)_2_) [7]. While Ca(OH)_2_ is a commonly used intracanal medicament, it demonstrates limited antimicrobial activity and lacks residual effects [8]. Furthermore, clinical studies suggest its efficacy falls below that of TAP [9,10,11]. A recent trial comparing mTAP (with cefaclor) to Ca(OH)_2_ found no significant difference in outcomes at three years [12]. However, a separate clinical trial using a lower concentration of TAP (0.1 mg/mL) revealed a significantly higher residual bacterial load compared to a higher concentration (1 g/mL) and Ca(OH)_2_ paste [13]. This concerning finding led to the discontinuation of the low-concentration TAP group due to early clinical failures. These findings highlight the need for improved intracanal medicaments that effectively manage bacterial infections while supporting regeneration in REP procedures.

Augmentin has shown efficacy against microorganisms cultured from such endodontic infections [14,15]. However, its use can be limited by potential discoloration of the tooth. TAP offers broad-spectrum antimicrobial activity but carries the risk of dentin staining the tooth [16]. The development of mTAP, which explores alternative antibiotics to replace minocycline, potentially promotes host cell function while maintaining effective antimicrobial action without tooth discoloration [17].

A key consideration when employing antibiotics in REP is balancing their effectiveness against bacteria with minimal adverse effects on stem cells, which is crucial for pulp–dentin complex regeneration [18]. High antibiotic concentrations are potentially more potent against bacteria and can impair stem cell viability. Biocompatible scaffolds loaded with antibiotics offer a promising solution, delivering sustained drug release within the root canal system to eliminate infection while preserving cell viability [19,20].

Hydrogels, nanogels, and chitosan scaffolds are materials explored for sustained and biocompatible medication delivery in REPs. Chitosan has garnered significant research interest due to its physicochemical and biological properties. These properties limit bacterial growth and enhance bioadherence and cell affinity, making chitosan a compelling candidate for antibiotic delivery in REPs [21,22,23].

This study investigated the biocompatibility and antibacterial efficacy potential of chitosan–gelatin (CH-G) scaffolds loaded with slow-releasing antibiotic formulations used in REPs. Therefore, the null hypothesis was that there is no significant difference in the proliferation, viability, or antibacterial activity potential of CH-G scaffolds loaded with different antibiotic formulations (augmentin, mTAP) or Ca(OH)_2_.

## 2. Materials and Methods

### 2.1. Ethical Approval

This study was approved by the Institutional Review Board (project number E-20-4887).

### 2.2. Fabrication of CH-G Scaffolds

CH-G scaffolds were fabricated using a previously described freeze-drying method [24]. Briefly, 2% (*w*/*v*) chitosan (75–85% deacetylated, molecular weight 310,000–375,000; Sigma-Aldrich/Merck, Darmstadt, Germany) solution in 1% (*v*/*v*) acetic acid was prepared and centrifuged to remove undissolved residues. Subsequently, a 3% (*w*/*v*) gelatin (Sigma-Aldrich; St. Louis, MO, USA) solution was mixed with the chitosan solution in a 2:3 ratio. This homogeneous mixture was poured (500 mL) into customized molds (area: 5 mm^2^, thickness: 3 mm^2^). The samples were refrigerated at 4 °C, frozen at −80 °C for 4 h, and freeze-dried at −56 °C overnight to obtain 3D CH-G scaffolds.

### 2.3. Sterilization and Neutralization

The resulting scaffolds (diameter: 5 mm, thickness: 1 mm) were sterilized using gamma radiation at 25 kGy. All scaffolds were neutralized by immersion in 10% NaOH, followed by washing with sterile water until neutrality was achieved.

### 2.4. Characterization of CH-G Scaffolds by Scanning Electron Microscopy (SEM)

The microstructure of the scaffolds was evaluated using JSM-6610LV scanning electron microscopy (SEM) (JEOL, Tokyo, Japan). Dried CH-G scaffolds were sputter-coated with gold after being mounted on an electric adhesive film. The size and structure of the scaffolds were examined using SEM, as previously described [24]. Pore size was estimated by measuring at least 30 pores from different cross-sections of three scaffolds using the SEM Control User Interface software (Version 3.11).

### 2.5. Preparation of Antibiotic Formulations

Stock solutions of augmentin and mTAP medicaments (10 mg/mL) were prepared by slowly dissolving equal amounts of each antibiotic powder in sterile water under continuous stirring within a sterile environment. Selected doses (0.1 mg/mL and 1.0 mg/mL) of each medicament (augmentin or mTAP) were prepared by diluting the stock solutions. Ca(OH)_2_ medicament was prepared by mixing pure Ca(OH)_2_ powder (Somatco, Riyadh, Saudi Arabia) with water at a concentration of 10 mg/mL [25].

### 2.6. Antibiotics Loading and Incubation

Dry CH-G scaffolds were placed in a 24-well plate and immersed in 1 mL of one of the five experimental medicaments or the control solution (phosphate-buffered saline, PBS) for overnight incubation at 37 °C. The six experimental groups were as follows:

Group 1: CH-G + 0.1 mg/mL augmentin;
Group 2: CH-G + 1 mg/mL augmentin;
Group 3: CH-G + 0.1 mg/mL mTAP;
Group 4: CH-G + 1 mg/mL mTAP;
Group 5: CH-G + 10 mg/mL Ca(OH)_2_;
Control: CH-G + PBS.

Following incubation, the hydrated scaffolds were gently transferred to fresh PBS in 24-well plates and maintained in an incubator at 37 °C until further experimentation.

### 2.7. Release of Antibiotics from CH-G Scaffolds

The in vitro release profiles of augmentin and mTAP from the CH-G scaffolds were analyzed using ultraviolet (UV) spectrophotometry [26]. Briefly, scaffolds loaded with each antibiotic (0.33 mg/mL) were incubated in 5 mL of PBS (pH 7.2) at 37 °C for one week. At predetermined time points (1, 2, 4, and 6 h; 1, 2, 5, 6, and 7 days), 1 mL aliquots were withdrawn from each well and replaced with fresh PBS to maintain a constant volume. The withdrawn aliquots were centrifuged to remove any scaffold debris. The supernatants were then analyzed using UV spectrophotometry at the following wavelengths specific for each antibiotic as per the following equation:

$$=\frac{\sum_{t\to 0}^{t}M_{t}}{M_{Actual}}\times 100\%$$

*M* represents the mass of the antibiotic drug released at a specific time point during the measurement period. $M_{t}$ signifies the total amount of the antibiotic drug initially present within the scaffold material. *M* (actual) represents the release exponent, which mathematically describes the pattern of drug release over time.

### 2.8. Cell Culture and Seeding

Human mesenchymal stem cells (hMSCs) were used in this study. The isolation procedures for the hMSC line followed previously established protocols [27,28]. Cells were seeded onto 24-well plates at a density of 1 × 10^6^ cells per well. The culture medium consisted of low-glucose Dulbecco’s Modified Eagle’s Medium (DMEM; Gibco/Thermo Fisher Scientific, Waltham, MA, USA) supplemented with 10% fetal bovine serum (FBS; Hyclone/Cytiva, Marlborough, MA, USA), 0.25 mol/L D-glucose, 0.004 mol/L L-glutamine, 0.006 mol/L sodium pyruvate, 100 U/mL penicillin–streptomycin, and non-essential amino acids (Gibco/Thermo Fisher Scientific). Cells were maintained in a humidified incubator set at 37 °C with 5% CO_2_. Following seeding, hMSCs were incubated for 24 h to facilitate attachment and growth to 80–90% confluence. All experiments were performed in triplicate with a sample size of *n* = 4 per replicate, resulting in a total of *n* = 12 samples per group.

### 2.9. Cell Viability and Proliferation

CH-G scaffolds loaded with the experimental and control solutions were placed over hMSCs seeded in 24-well plates at a × 10^6^ cells/well. Density cell viability was assessed over 3 days using the Alamar Blue assay (AbD Serotec, Kidlington, UK) according to the manufacturer’s instructions [1]. Briefly, cells were incubated for 24 h to allow attachment. Scaffolds were added and incubated for 1, 2, or 3 days. At each time point, 10% Alamar Blue was added to each well and incubated for 2 h. Fluorescence intensity (excitation/emission 530/590 nm) was measured using a fluorescence reader (BioTek, Winooski, VT, USA).

Cell proliferation and attachment were evaluated using SEM (JSM-6360LV SEM; Jeol, Tokyo, Japan). After 3 days, cells were washed with PBS, fixed with 2.5% glutaraldehyde, washed again, and dehydrated. Images were obtained at 5000× magnification.

### 2.10. Live/Dead Imaging

Acridine orange (AO) and ethidium bromide (EB) staining were used to assess apoptosis and necrosis after 72 h of medicament application. Media was removed, and cells were washed and stained for 2 min with a solution containing 100 μg/mL each of AO and EB (Sigma-Aldrich/Merck, Darmstadt, Germany). Cells were imaged using a Nikon Eclipse fluorescence microscope (Nikon, Tokyo, Japan). Live cells appear green with AO staining, while necrotic cells appear red with EB staining.

### 2.11. E. faecalis Strain and Biofilm Formation

Biofilms were prepared following a previously described protocol [29]. Briefly, a standardized suspension of *E. faecalis* (ATCC 29212) was cultured in brain heart infusion (BHI) broth at 37 °C for 24 h. Sterilized dentin discs were placed in a 96-well plate with the pulpal side facing upwards. Each well received 3 mL of *E. faecalis* suspension adjusted to 1 × 10^8^ CFU/mL. The plates were incubated at 37 °C for 3 weeks in 100% humidity to allow biofilm formation. The culture medium was replenished twice weekly to maintain consistent bacterial growth.

### 2.12. Treatment of Infected Specimens

Experimental scaffolds (*n* = 4 per group, *n* = 12 total with triplicate experiments) were placed over the dentin discs. The discs were then incubated at 37 °C and 100% humidity for 7 days. Untreated dentin discs with established biofilms (*n* = 10) were exposed to a sterile saline solution, which served as the positive control for biofilm viability. Biofilm viability on these control discs was verified using confocal laser scanning microscopy (CLSM; Nikon C2Si, Nikon, Tokyo, Japan) and SEM.

### 2.13. Assessment of E. faecalis Viability

After 7 days of treatment, the metabolic activity of *E. faecalis* biofilms was assessed using the MTT assay [30]. Scaffolds were removed from each well, and the dentin discs were washed with 5 mL of sterile PBS to remove residual medicaments. Subsequently, 150 μL of PBS and 50 μL of MTT solution (Millipore Sigma/Merck, Darmstadt, Germany) were added to each well. The plates were incubated for 2 h at 37 °C in a light-protected environment to allow formazan crystal formation by metabolically active bacteria within the biofilms. The absorbance of the resulting solution was measured at 570 nm using a microplate reader (BioTek Synergy II; BioTek Inc., Winooski, VT, USA). Higher absorbance values indicate greater metabolic activity and more viable bacteria within the biofilms. Selected specimens from each group were processed for SEM analysis using a JSM-6360LV SEM (JEOL, Tokyo, Japan).

Bacterial viability within the biofilms was assessed using the Live/Dead BacLight Bacterial Viability Kit (Molecular Probes/Thermo Fisher Scientific, Waltham, MA, USA) according to the manufacturer’s protocol. Briefly, samples were stained with a mixture of SYTO 9 green, fluorescent nucleic acid stain (live cells) and propidium iodide red fluorescent nucleic acid stain (dead cells). Confocal laser scanning microscopy (CLSM) was then performed to visualize the stained biofilms. Biofilm images obtained via CLSM were processed and quantified using Fiji software (Fiji, ImageJ, Wayne Rasband National Institutes of Health, Version 2.9.0), an open-source platform widely used for biological image analysis. The software was used to measure the fluorescence intensity of both SYTO 9 (green) and propidium iodide (red) within the biofilms. The ratio of red fluorescence intensity to total fluorescence intensity (red + green) served as a measure of the proportion of dead cells within each sample. This ratio was then converted to a percentage to determine the relative viability of the bacterial population within the biofilms.

Statistical Analyses

Statistical analyses were performed using IBM SPSS Statistics software (version 28; IBM Corp., Armonk, NY, USA). Data on cell proliferation, differentiation, and antibacterial activity (MTT assay) were assessed for normality and homogeneity of variance. Normally distributed data with equal variances were analyzed using one-way analysis of variance (ANOVA) followed by Tukey’s post hoc test for multiple comparisons between groups. Appropriate non-parametric tests were employed for data with unequal variances or non-normal distribution. All statistical tests were two-tailed, and a significance level of α = 0.05 was used.

## 3. Results

### 3.1. Microstructure of CH-G Scaffolds

**Figure 1:**
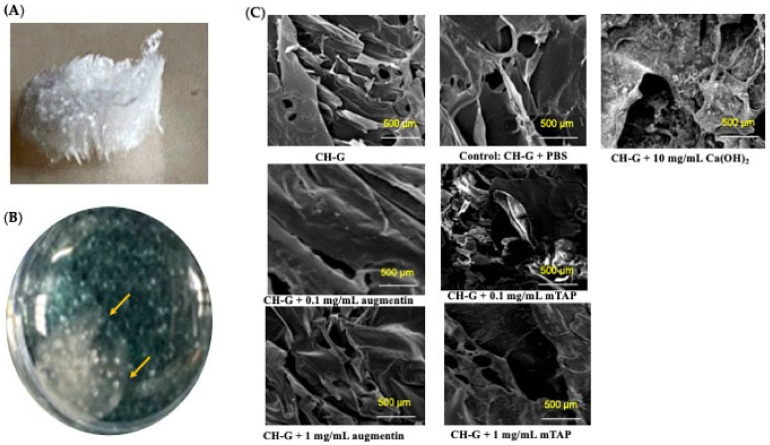
(**A**) Three-dimensional representation of dry chitosan–gelatin scaffold. (**B**) Image of the hydrated and neutralized scaffold in a 24-well plate. (**C**) SEM images of the dry scaffold and immersed scaffold in experimental groups and the control. Note: The dry scaffold CH-G is highly porous with an interconnected pore structure, unlike the immersed scaffold in different antibiotics, which shows reduced pore size.

Visual inspection revealed a sponge-like structure for the lyophilized CH-G scaffolds (Figure 1A). Upon hydration and neutralization, the scaffolds became colorless, soft, flexible, and gelatinous (Figure 1B). SEM analysis of the dry scaffolds demonstrated a highly porous structure with interconnected spherical pores ranging in size from 31 to 69 µm (mean diameter: 56 µm) (Figure 1C). Interestingly, scaffolds loaded with antibiotics exhibited some swelling and pore expansion.

### 3.2. Differential Release of Medicaments from the CH-G Scaffold

**Figure 2:**
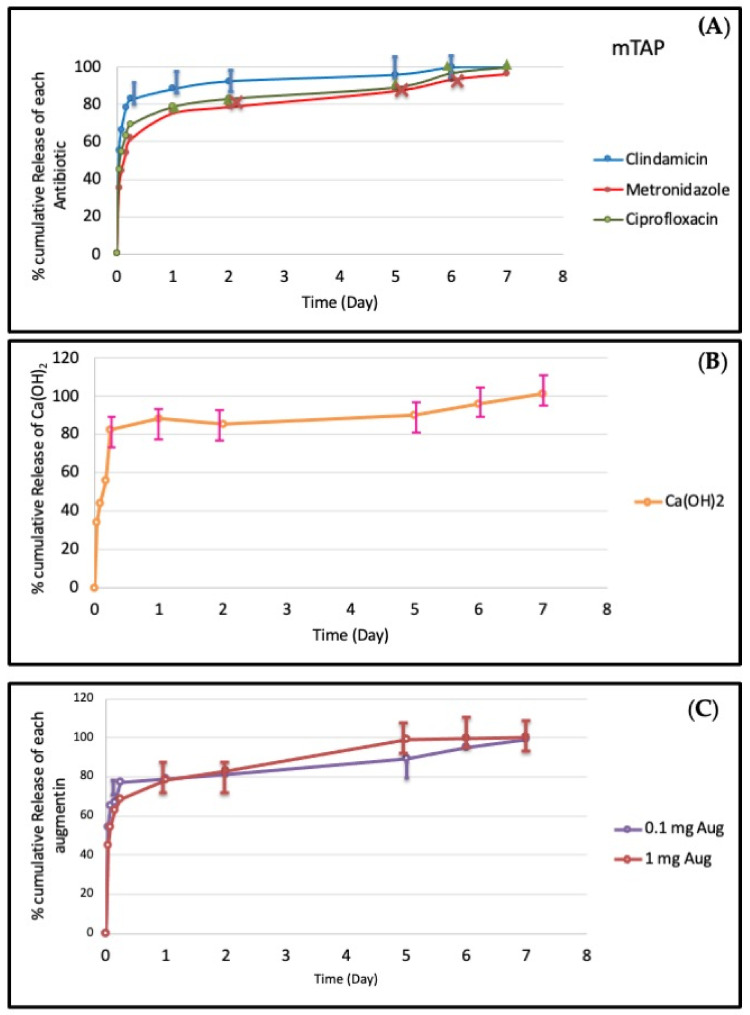
(**A**) Cumulative release (%) of antibiotic (metronidazole, ciprofloxacin, minocycline, and clindamycin) at 0.33 mg/mL from chitosan–gelatin scaffold (*n* = 4 in triplicate). (**B**) Cumulative release (%) of Ca(OH) at (10 mg) from chitosan–gelatin scaffold (*n* = 4 in triplicate). (**C**) Cumulative release (%) of augmentin from chitosan–gelatin scaffold (*n* = 4 in triplicate).

The release profiles of various medicaments from the CH-G scaffolds are presented in Figure 2. All medicaments displayed an initial burst release within the first hour, followed by a sustained release phase reaching a plateau after 7 days. By the 4 h mark, over 88% of clindamycin had been released from the scaffold, followed by metronidazole (77%) and ciprofloxacin (68%). Similarly, Ca(OH)_2_ exhibited a 40% initial burst release within the first hour. Augmentin displayed dose-dependent release, with 0.1 mg and 1 mg formulations achieving approximately 40% and 60% release in the first hour. Notably, all medicaments surpassed 80% cumulative release and exhibited a more linear release pattern after the first day, reaching nearly 100% release by day seven.

### 3.3. Augmentin Enhances Cell Proliferation, While Augmentin and mTAP Improve Viability

**Figure 3:**
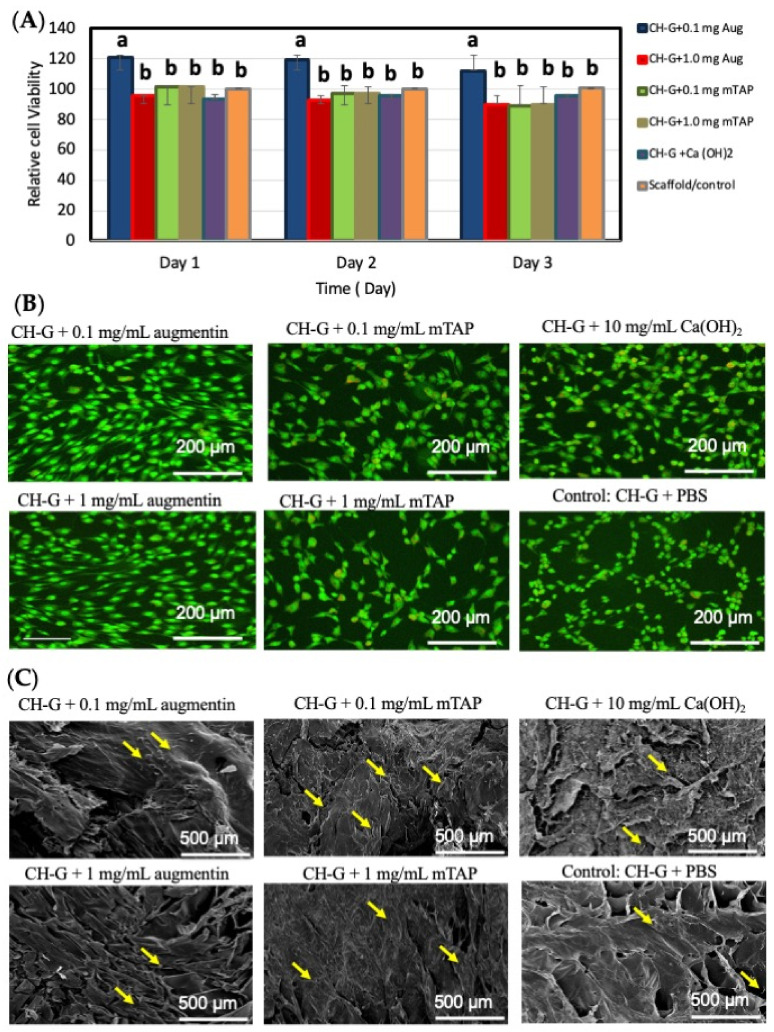
(**A**) Results of proliferation (Alamar Blue assay). Different lowercase letters indicate statistical significance when compared with control at each time point. Error bars indicate standard deviations. “bar labeled with different superscript letter a indicate significant different from the others labeled with b”. (**B**) Representative fluorescent microscope images (20x) of hMSCs treated with experimental groups showing live (green) and dead (red) stain. (Scale bar = 200 μm). (**C**) SEM image analysis of hMSCs after three days exposed to experimental groups and the control. Yellow arrows indicate cells. (Scale bar = 500 µm).

Cell proliferation was significantly greater (*p* < 0.05) in the 0.1 mg/mL augmentin group compared to all other groups at all time points (Figure 3A). This finding was further supported by live/dead staining, which revealed a significantly higher proportion of viable cells (green stain) in the 0.1 mg/mL augmentin group compared to other groups (Figure 3B).

SEM analysis revealed distinct morphological structures across treatment groups. Cells cultured with only the scaffold appeared flat, unattached, and less spread out, lacking prominent cytoplasmic extensions. In contrast, cells treated with augmentin displayed a rounded structure with a wider distribution within the scaffold. Cells in this group exhibited a more spindle-shaped appearance. Conversely, the mTAP group showed cells with multiple thin cytoplasmic extensions, appearing more abundant and rounded at the edges, with good attachment to the scaffold. Cells in the Ca(OH)_2_ group displayed detached cells with visible cytoplasmic extensions (Figure 3C).

### 3.4. mTAP and Augmentin Exhibit Antimicrobial Activity

**Figure 4:**
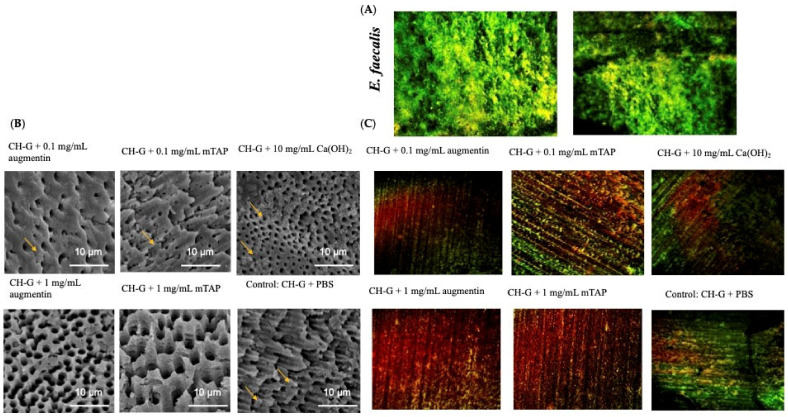
(**A**) 21-day-old *E. faecalis* biofilm on dentin slices. (**B**) Scanning electron microscopic images (×1000) of 21-day-old *E. faecalis* biofilm on dentin slices treated with different medicaments for 7 days. Yellow arrowheads indicate bacterial rods of *E. faecalis*. (**C**) Representative confocal microscopy showing live (green) and dead (red) bacterial cells on the dentin surface after seven days of treatment with medicaments.

CLSM confirmed the presence of intact E. faecalis biofilms (Figure 4A). As shown in Table 1, the 1.0 mg/mL augmentin and mTAP groups displayed a significant reduction in bacterial viability compared to positive controls (*p* < 0.05). SEM analysis after seven days of treatment revealed a complete absence of bacterial biofilm on the surfaces of dentin discs in the 1.0 mg/mL augmentin and 1.0 mg/mL mTAP groups (Figure 4B). CLSM corroborated these findings (Figure 4C). Residual biofilm structures were observed on the dentin surface in the control, 0.1 mg/mL mTAP, and Ca(OH)_2_ groups. Table 2 indicates a higher proportion of dead cells within the 1.0 mg/mL mTAP group biofilms than other treatments.

## 4. Discussion

This study investigated the potential of CH-G scaffolds loaded with various antibiotic formulations for their use in REPs. The choice of CH-G as the scaffold material relates to its well-established biocompatibility and suitability for controlled drug delivery [31]. Scaffolds require a highly porous structure with interconnected pores for effective tissue engineering. It facilitates a favorable biological environment for cell attachment, proliferation, and tissue regeneration [32]. The CH-G scaffolds employed in this study addressed this crucial aspect with their large pore size, demonstrably promoting cell migration, as evidenced in previous research [21]. Furthermore, CH-G scaffolds can effectively absorb various antibiotics through passive absorption and diffusion [33,34,35,36].

Our findings confirm the ability of CH-G scaffolds to support the sustained release of antibiotics, potentially creating a localized environment that promotes both effective disinfection and tissue regeneration. SEM analysis confirmed that the solutions did not compromise scaffold integrity. It offers several advantages over traditional endodontic treatments. Sustained antibiotic release from the scaffold provides prolonged antibacterial activity within the root canal, thus potentially reducing the risk of reinfection compared to bolus delivery methods. Additionally, the biocompatible nature of CH-G minimizes the potential for adverse tissue reactions compared to some conventional medicaments [37]. Previous research by Thein-Han et al. (2009) demonstrated a slow degradation profile for chitosan–gelatin scaffolds in a simulated physiological environment over a 28-day period. However, it is important to acknowledge that their study also observed a significant and rapid degradation phase during the initial 7 days [24].

The CH-G scaffold combines the beneficial properties of its constituent materials, potentially creating a microenvironment conducive to tissue regeneration. Gelatin, a component of the scaffold, is known to promote cellular activity, including adhesion, differentiation, and proliferation [24]. Chitosan, on the other hand, contributes its inherent antibacterial properties [23]. Additionally, porous chitosan structures have been shown to enhance chemotaxis, the regenerative potential of dental pulp cells, and odontoblastic differentiation [38]. This synergistic interaction between the scaffold’s components may establish a microenvironment that is not only biocompatible, as demonstrated in our study, but also actively promotes tissue regeneration. While the SEM analysis confirms that the antibiotic solutions did not compromise the overall integrity of the scaffold itself, it is important to acknowledge that this analysis does not necessarily guarantee the absence of any mechanical stress on the cells. Future studies could explore the potential influence of scaffold stiffness on cell behavior through methods like incorporating scaffolds with varying stiffness or employing techniques to measure cellular response to mechanical cues.

The immortalized human bone marrow MSCs utilized in the present study demonstrated cellular and molecular phenotypes, like primary hMSCs [27,28]. Their multipotency and differentiation capabilities are comparable to dental pulp stem cells, making them relevant to REPs [39,40,41,42]. Furthermore, research suggests that bone marrow stem cells can induce odontoblasts and enhance dentin mineralization [43,44]. Dental pulp cells and bone marrow share similar genetic mechanisms in osteoblasts and odontoblast osteogenic differentiation [45].

The rising prevalence of antibiotic resistance in oral bacteria underscores the need for exploring broad-spectrum antibiotics. Augmentin, a first-line antibiotic for treating spreading endodontic infections, demonstrated efficacy against bacterial biofilms in the present study at 1 mg/mL. It aligns with a clinical study where augmentin was the only antibiotic effective against all facultative and anaerobic bacteria isolated from patients with acute endodontic abscesses [46]. Furthermore, cell viability and proliferation assays indicated increased cell survival following treatment with augmentin compared to other agents, corroborating a previous study reporting a 100% increase in cell survival rate with low-dose augmentin [47]. A successful regenerative endodontic case using augmentin paste for root canal disinfection has also been reported [48]. However, routine clinical use of augmentin as an intracanal medication is limited due to concerns regarding penicillin allergies [49].

The mTAP group emerged as a promising option, demonstrating significant antibacterial activity against biofilms without compromising cell viability. Recent in vitro studies support mTAP’s effectiveness as an antimicrobial against biofilms [23]. Notably, a study showed that mTAP exhibited the highest antimicrobial activity against *E. faecalis* biofilms within deep intratubular dentin compared to TAP and Ca(OH)_2_ [50]. The inclusion of clindamycin in mTAP offers several benefits. Clindamycin exhibits potent activity against Gram-positive bacteria and obligate facultative anaerobes and possesses desirable properties for promoting bone growth [51]. Furthermore, it has superior bone penetration compared to commonly used antibiotics like penicillin [52].

While Ca(OH)_2_ displayed some biocompatibility (no cytotoxicity observed), its incorporation within the scaffold altered cell structure and did not achieve complete biofilm eradication. These findings align with previous research that suggests the limitations of Ca(OH)_2_ for REPs [39]. Several factors might contribute to the observed inefficacy of Ca(OH)_2_ in this context. Firstly, compared to other antibiotic combinations like TAP or DAP, Ca(OH)_2_ exhibits lower efficacy against established biofilms [9,10,11]. Secondly, Ca(OH)_2_ may not be optimal for targeting common endodontic pathogens associated with persistent infections [11]. Finally, limitations in its solubility, diffusibility, and dentin-buffering capacity, even at higher concentrations within the scaffold, might restrict its effectiveness [53]. These limitations highlight the need to explore alternative antibiotic formulations within CH-G scaffolds for optimal biofilm eradication during REPs.

This study employed two methods, the MTT assay and CLSM, to assess the reduction in bacterial burden. This approach comprehensively evaluated the antibacterial efficacy of various agents released from the CH-G scaffold on the dentin surface and within the dentinal tubules. The MTT assay selection aligns with previous studies utilizing the same model to evaluate the metabolic activity of *E. faecalis* and its biofilm formation [30]. The MTT assay offers several advantages, including rapid results, ease of use, and the ability to assess cell density in small cultures [54]. Even though this study was carried out against a single microorganism, it is the first to examine the potential of CH-G scaffolds loaded with augmentin and mTAP.

The study was conducted entirely in vitro, which inherently limits its translation to the complex biological environment of a real REP procedure. Alternative cell seeding methods, such as direct seeding or transwell migration assays, are needed to better simulate cell migration and population within the 3D scaffold structure. Future studies involving animal models or even human tissues will be necessary to assess the true effectiveness of these scaffolds in a more clinically relevant setting. The use of a single bacterial species and individual cells, instead of a more complex tissue model, represents a limitation. This approach does not fully capture the intricate interactions between various cell types and bacterial communities present in an infected tooth.

This study demonstrates the promising potential of CH-G scaffolds loaded with antibiotics for use in REPs. However, further research is necessary to fully translate this potential into clinical applications. Future studies should investigate strategies to optimize the loading and release kinetics of various antibiotics within the CH-G scaffolds. This could involve exploring different formulations, scaffold properties, or release mechanisms to tailor them to specific clinical needs and target bacteria. Expanding the research to investigate the efficacy against multispecies biofilms, which are prevalent in infected root canals, would provide a more realistic picture of the potential clinical effectiveness. In vivo studies are crucial to validate the long-term efficacy and safety of these scaffolds in a more realistic physiological environment. This will allow for a more comprehensive evaluation of their potential benefits and limitations in a clinical setting. Further investigation into the ability of the CH-G scaffolds to induce mineral matrix deposition, a key aspect of dentin regeneration, is warranted. Explorations into the combined effects of different antibiotic formulations within the scaffolds and their potential synergistic benefits for biofilm eradication hold promise. This could lead to more effective treatment strategies for complex infections. By addressing these areas, future research can refine the design and application of CH-G scaffolds for REPs, ultimately leading to improved clinical outcomes.

## 5. Conclusions

This study suggests that CH-G scaffolds loaded with specific antibiotics, particularly augmentin and mTAP at a concentration of 1 mg/mL, offer a promising approach for endodontic regeneration. Their biocompatibility, antibacterial efficacy, and ability to promote cell attachment provide a foundation for developing novel therapeutic strategies in this field.

## Figures and Tables

**Table 1:** *E. Faecalis* viability measured via MTT assay at 7 days treatment.

Group	M ± SD	95% Confidence Interval	Difference from Positive Control
Dentin discs + *E. Faecalis *(Positive control)	0.41 ± 0.18	0.13 to 0.69	
Dentin discs/CH-G alone (negative control)	0.37 ± 0.13	0.16 to 0.58	*p* = 0.999
Dentin discs/CH-G +Ca (OH)_2_ + *E. Faecalis*	0.30 ± 0.15	0.05 to 0.55	*p* = 0.895
Dentin discs/CH-G + 0.1 mg/mL augmentin + *E. Faecalis*	0.29 ± 0.11	0.11 to 0.46	*p* = 0.846
Dentin discs/CH-G + 1 mg/mL augmentin + *E. Faecalis*	0.03 ± 0.05	−0.09 to 0.03	*p* < 0.001 *
Dentin discs/CH-G + 0.1 mg mTAP + *E. Faecalis*	0.05 ± 0.20	−0.20 to 0.30	*p* = 0.008 *
Dentin discs/CH-G + 1.0 mg mTAP + *E. Faecalis*	0.01 ± 0.04	−0.02 to 0.08	*p* = 0.000 *

* Indicates statistically significant value from the positive control (*p* < 0.05). *p*-values are from the Tukey HSD post hoc test.

**Table 2:** Median and range values of dead cells of the positive control and experimental groups after 7 days of treatment.

Group	Median (Range) *
Dentin discs + *E. Faecalis *(Positive control)	25.4 (13.85–25.8) ^a^
Dentin discs/CH-G alone (negative control)	56.09 (27.9–56.4) ^b^
Dentin discs/CH-G +Ca (OH)_2_ + *E. Faecalis*	65.35 (40.1–65.2) ^c^
Dentin discs/CH-G + 0.1 mg/mL augmentin + *E. Faecalis*	80.27 (59.57–75.35) ^d^
Dentin discs/CH-G + 1 mg/mL augmentin + *E. Faecalis*	83.59 (83.208–88.8) ^d^
Dentin discs/CH-G + 0.1 mg mTAP + *E. Faecalis*	81.59 (41.56–85.9) ^d^
Dentin discs/CH-G + 1.0 mg mTAP + *E. Faecalis*	91.52(83.1–94.87) ^e^

* Data labeled with different superscript letters; a, b, c, d, e in the table are significantly different from each other (*p* < 0.05).

## Data Availability

The data of this study are available from the corresponding authors upon reasonable request.

## References

[1] Trope M. (2010). Treatment of the immature tooth with a nonvital pulp and apical periodontitis. Dent. Clin. N. Am..

[2] Diogenes A., Ruparel N.B., Shiloah Y., Hargreaves K.M. (2016). Regenerative endodontics: A way forward. J. Am. Dent. Assoc..

[3] Thelen D.S., Trovik T.A., Bårdsen A. (2011). Impact of traumatic dental injuries with unmet treatment need on daily life among Albanian adolescents: A case-control study. Dent. Traumatol..

[4] Duncan H.F., Cooper P.R., Smith A.J. (2019). Dissecting dentine-pulp injury and wound healing responses: Consequences for regenerative endodontics. Int. Endod. J..

[5] Almutairi W., Yassen G.H., Aminoshariae A., Williams K.A., Mickel A. (2019). Regenerative Endodontics: A Systematic Analysis of the Failed Cases. J. Endod..

[6] Fouad A.F. (2020). Contemporary Microbial and Antimicrobial Considerations in Regenerative Endodontic Therapy. J. Endod..

[7] American Association of Endodontists (AAE) (2021). Clinical Considerations for a Regenerative Procedure. https://www.aae.org/specialty/wp-content/uploads/sites/2/2021/08/ClinicalConsiderationsApprovedByREC062921.pdf.

[8] Sathorn C., Parashos P., Messer H. (2007). Antibacterial efficacy of calcium hydroxide intracanal dressing: A systematic review and meta-analysis. Int. Endod. J..

[9] Arruda M.E.F., Neves M.A.S., Diogenes A., Mdala I., Guilherme B.P.S., Siqueira J.F., Rôças I.N. (2018). Infection Control in Teeth with Apical Periodontitis Using a Triple Antibiotic Solution or Calcium Hydroxide with Chlorhexidine: A Randomized Clinical Trial. J. Endod..

[10] Chrepa V., Joon R., Austah O., Diogenes A., Hargreaves K.M., Ezeldeen M., Ruparel N.B. (2020). Clinical Outcomes of Immature Teeth Treated with Regenerative Endodontic Procedures-A San Antonio Study. J. Endod..

[11] Casey S.M., Fox D., Duong W., Bui N., Latifi N., Ramesh V., Podborits E., Flake N.M., Khan A.A., Gibbs J.L. (2022). Patient Centered Outcomes among a Cohort Receiving Regenerative Endodontic Procedures or Apexification Treatments. J. Endod..

[12] Al-Qudah A., Almomani M., Hassoneh L., Awawdeh L. (2023). Outcome of Regenerative Endodontic Procedures in Nonvital Immature Permanent Teeth Using 2 Intracanal Medications: A Prospective Randomized Clinical Study. J. Endod..

[13] Fouad A.F., Diogenes A.R., Torabinejad M., Hargreaves K.M. (2022). Microbiome changes during regenerative endodontic treatment using different methods of disinfection. J. Endod..

[14] Jungermann G.B., Burns K., Nandakumar R., Tolba M., Venezia R.A., Fouad A.F. (2011). Antibiotic resistance in primary and persistent endodontic infections. J. Endod..

[15] Baumgartner J.C., Xia T. (2003). Antibiotic susceptibility of bacteria associated with endodontic abscesses. J. Endod..

[16] Diogenes A.R., Ruparel N.B., Teixeira F.B., Hargreaves K.M. (2014). Translational science in disinfection for regenerative endodontics. J. Endod..

[17] Karczewski A., Feitosa S.A., Hamer E.I., Pankajakshan D., Gregory R.L., Spolnik K.J., Bottino M.C. (2018). Clindamycin-modified Triple Antibiotic Nanofibers: A Stain-free Antimicrobial Intracanal Drug Delivery System. J. Endod..

[18] Trevino E.G., Patwardhan A.N., Henry M.A., Perry G., Dybdal-Hargreaves N., Hargreaves K.M., Diogenes A. (2011). Effect of irrigants on the survival of human stem cells of the apical papilla in a platelet-rich plasma scaffold in human root tips. J. Endod..

[19] Bottino M.C., Kamocki K., Yassen G.H., Platt J.A., Vail M.M., Ehrlich Y., Spolnik K.J., Gregory R.L. (2013). Bioactive nanofibrous scaffolds for regenerative endodontics. J. Dent. Res..

[20] Palasuk J., Kamocki K., Hippenmeyer L., Platt J.A., Spolnik K.J., Gregory R.L., Bottino M.C. (2014). Bimix antimicrobial scaffolds for regenerative endodontics. J. Endod..

[21] Sharma P.K., Halder M., Srivastava U., Singh Y. (2019). Antibacterial PEG-Chitosan Hydrogels for Controlled Antibiotic/Protein Delivery. ACS Appl. Bio Mater..

[22] Bellamy C., Shrestha S., Torneck C., Kishen A. (2016). Effects of a Bioactive Scaffold Containing a Sustained Transforming Growth Factor-β1-releasing Nanoparticle System on the Migration and Differentiation of Stem Cells from the Apical Papilla. J. Endod..

[23] Aksel H., Mahjour F., Bosaid F., Calamak S., Azim A.A. (2020). Antimicrobial Activity and Biocompatibility of Antibiotic-Loaded Chitosan Hydrogels as a Potential Scaffold in Regenerative Endodontic Treatment. J. Endod..

[24] Thein-Han W.W., Saikhun J., Pholpramoo C., Misra R.D., Kitiyanant Y. (2009). Chitosan-gelatin scaffolds for tissue engineering: Physico-chemical properties and biological response of buffalo embryonic stem cells and transfectant of GFP-buffalo embryonic stem cells. Acta Biomater..

[25] AlGazlan A.S., Auda S.H., Balto H., Alsalleeh F. (2022). Antibiofilm Efficacy of Silver Nanoparticles Alone or Mixed with Calcium Hydroxide as Intracanal Medicaments: An Ex-Vivo Analysis. J. Endod..

[26] Torshabi M., Nojehdehian H., Tabatabaei F.S. (2017). In vitro behavior of poly-lactic-co-glycolic acid microspheres containing minocycline, metronidazole, and ciprofloxacin. J. Investig. Clin. Dent..

[27] Simonsen J.L., Rosada C., Serakinci N., Justesen J., Stenderup K., Rattan S.I., Jensen T.G., Kassem M. (2002). Telomerase expression extends the proliferative life-span and maintains the osteogenic potential of human bone marrow stromal cells. Nat. Biotechnol..

[28] Abdallah B.M., Haack-Sørensen M., Burns J.S., Elsnab B., Jakob F., Hokland P., Kassem M. (2005). Maintenance of differentiation potential of human bone marrow mesenchymal stem cells immortalized by human telomerase reverse transcriptase gene despite of extensive proliferation. Biochem. Biophys. Res. Commun..

[29] Balto H., Bukhary S., Al-Omran O., BaHammam A., Al-Mutairi B. (2020). Combined Effect of a Mixture of Silver Nanoparticles and Calcium Hydroxide against Enterococcus faecalis Biofilm. J. Endod..

[30] He Z., Liang J., Zhou W., Xie Q., Tang Z., Ma R., Huang Z. (2016). Effect of the quorum-sensing luxS gene on biofilm formation by Enterococcus faecalis. Eur. J. Oral. Sci..

[31] Tan H., Wu J., Lao L., Gao C. (2009). Gelatin/chitosan/hyaluronan scaffold integrated with PLGA microspheres for cartilage tissue engineering. Acta Biomater..

[32] Cohen S., Baño M.C., Cima L.G., Allcock H.R., Vacanti J.P., Vacanti C.A., Langer R. (1993). Design of synthetic polymeric structures for cell transplantation and tissue engineering. Clin. Mater..

[33] Wells C.M., Beenken K.E., Smeltzer M.S., Courtney H.S., Jennings J.A., Haggard W.O. (2018). Ciprofloxacin and Rifampin Dual Antibiotic-Loaded Biopolymer Chitosan Sponge for Bacterial Inhibition. Mil. Med..

[34] Parker A.C., Jennings J.A., Bumgardner J.D., Courtney H.S., Lindner E., Haggard W.O. (2013). Preliminary investigation of crosslinked chitosan sponges for tailorable drug delivery and infection control. J. Biomed. Mater. Res. B Appl. Biomater..

[35] Noel S.P., Courtney H.S., Bumgardner J.D., Haggard W.O. (2010). Chitosan sponges to locally deliver amikacin and vancomycin: A pilot in vitro evaluation. Clin. Orthop. Relat. Res..

[36] Stinner D.J., Noel S.P., Haggard W.O., Watson J.T., Wenke J.C. (2010). Local antibiotic delivery using tailorable chitosan sponges: The future of infection control?. J. Orthop. Trauma.

[37] Sungkhaphan P., Thavornyutikarn B., Kaewkong P., Pongkittiphan V., Pornsuwan S., Singhatanadgit W., Janvikul W. (2021). Antibacterial and osteogenic activities of clindamycin-releasing mesoporous silica/carboxymethyl chitosan composite hydrogels. R. Soc. Open Sci..

[38] Amir L.R., Suniarti D.F., Utami S., Abbas B. (2014). Chitosan as a potential osteogenic factor compared with dexamethasone in cultured macaque dental pulp stromal cells. Cell Tissue Res..

[39] Shi S., Robey P.G., Gronthos S. (2001). Comparison of human dental pulp and bone marrow stromal stem cells by cDNA microarray analysis. Bone.

[40] Murakami M., Hayashi Y., Iohara K., Osako Y., Hirose Y., Nakashima M. (2015). Trophic Effects and Regenerative Potential of Mobilized Mesenchymal Stem Cells From Bone Marrow and Adipose Tissue as Alternative Cell Sources for Pulp/Dentin Regeneration. Cell Transpl..

[41] Huang G.T., Gronthos S., Shi S. (2009). Mesenchymal stem cells derived from dental tissues vs. those from other sources: Their biology and role in regenerative medicine. J. Dent. Res..

[42] Al-Nbaheen M., Vishnubalaji R., Ali D., Bouslimi A., Al-Jassir F., Megges M., Prigione A., Adjaye J., Kassem M., Aldahmash A. (2013). Human stromal (mesenchymal) stem cells from bone marrow, adipose tissue and skin exhibit differences in molecular phenotype and differentiation potential. Stem Cell Rev. Rep..

[43] Yamada Y., Fujimoto A., Ito A., Yoshimi R., Ueda M. (2006). Cluster analysis and gene expression profiles: A cDNA microarray system-based comparison between human dental pulp stem cells (hDPSCs) and human mesenchymal stem cells (hMSCs) for tissue engineering cell therapy. Biomaterials.

[44] Batouli S., Miura M., Brahim J., Tsutsui T.W., Fisher L.W., Gronthos S., Robey P.G., Shi S. (2003). Comparison of stem-cell-mediated osteogenesis and dentinogenesis. J. Dent. Res..

[45] Gaus S., Li H., Li S., Wang Q., Kottek T., Hahnel S., Liu X., Deng Y., Ziebolz D., Haak R. (2021). Shared Genetic and Epigenetic Mechanisms between the Osteogenic Differentiation of Dental Pulp Stem Cells and Bone Marrow Stem Cells. Biomed. Res. Int..

[46] Khemaleelakul S., Baumgartner J.C., Pruksakorn S. (2002). Identification of bacteria in acute endodontic infections and their antimicrobial susceptibility. Oral Surg. Oral Med. Oral Pathol. Oral Radiol. Endodontol..

[47] Ruparel N.B., Teixeira F.B., Ferraz C.C., Diogenes A. (2012). Direct effect of intracanal medicaments on survival of stem cells of the apical papilla. J. Endod..

[48] Nosrat A., Li K.L., Vir K., Hicks M.L., Fouad A.F. (2013). Is pulp regeneration necessary for root maturation?. J. Endod..

[49] Macy E. (2014). Penicillin and beta-lactam allergy: Epidemiology and diagnosis. Curr. Allergy Asthma Rep..

[50] Cunha Neto M.A.D., Coêlho J.A., Pinto K.P., Cuellar M.R.C., Marcucci M.C., Silva E., Andrade F.B., Sassone L.M. (2021). Antibacterial Efficacy of Triple Antibiotic Medication With Macrogol (3Mix-MP), Traditional Triple Antibiotic Paste, Calcium Hydroxide, and Ethanol Extract of Propolis: An Intratubular Dentin Ex Vivo Confocal Laser Scanning Microscopic Study. J. Endod..

[51] Chow A.W. (2015). Infections of the oral cavity, neck, and head. Mandell, Douglas, and Bennett’s Principles and Practice of Infectious Diseases.

[52] Thabit A.K., Fatani D.F., Bamakhrama M.S., Barnawi O.A., Basudan L.O., Alhejaili S.F. (2019). Antibiotic penetration into bone and joints: An updated review. Int. J. Infect. Dis..

[53] Siqueira J.F., Lopes H.P. (1999). Mechanisms of antimicrobial activity of calcium hydroxide: A critical review. Int. Endod. J..

[54] Ata S.O., Akay C., Mumcu E., Erdonmez D. (2023). The Antibacterial and Antifungal Activity of Chlorhexidine Diacetate Incorporated into Acrylic Resins Used in Provisional Restorations. Acta Stomatol. Croat..

